# Microbial ecology of sand fly breeding sites: aging and larval conditioning alter the bacterial community composition of rearing substrates

**DOI:** 10.1186/s13071-022-05381-w

**Published:** 2022-07-26

**Authors:** Nayma Romo Bechara, Gideon Wasserberg, Kasie Raymann

**Affiliations:** University of North Carolina at Greensboro, Greensboro, NC USA

**Keywords:** Sand flies, Rearing substrates, Bacteria, Larva, Oviposition, Vector control

## Abstract

**Background:**

Sand flies vector several human pathogens, including *Leishmania* species, which cause leishmaniases. A leishmaniasis vaccine does not yet exist, so the most common prevention strategies involve personal protection and insecticide spraying. However, insecticides can impact non-target organisms and are becoming less effective because of the evolution of resistance. An alternative control strategy is the attract-and-kill approach, where the vector is lured to a lethal trap, ideally located in oviposition sites that will attract gravid females. Oviposition traps containing attractive microbes have proven successful for the control of some mosquito populations but have not been developed for sand flies. Gravid female sand flies lay their eggs in decomposing organic matter on which the larvae feed and develop. Studies have demonstrated that gravid females are particularly attracted to larval conditioned (containing eggs and larvae) and aged rearing substrates. An isolate-based study has provided some evidence that bacteria play a role in the attraction of sand flies to conditioned substrates. However, the overall bacterial community structure of conditioned and aged substrates and how they change over time has not been investigated.

**Methods:**

The goal of this study was to characterize the bacterial communities of rearing and oviposition substrates that have been shown to vary in attractiveness to gravid sand flies in previous behavioral studies. Using 16S rRNA amplicon sequencing we determined the bacterial composition in fresh, aged, and larval-conditioned substrates at four time points representing the main life-cycle stages of developing sand flies. We compared the diversity, presence, and abundance of taxa across substrate types and time points in order to identify how aging and larval-conditioning impact bacterial community structure.

**Results:**

We found that the bacterial communities significantly change within and between substrates over time. We also identified bacteria that might be responsible for attraction to conditioned and aged substrates, which could be potential candidates for the development of attract-and-kill strategies for sand flies.

**Conclusion:**

This study demonstrated that both aging and larval conditioning induce shifts in the bacterial communities of sand fly oviposition and rearing substrates, which may explain the previously observed preference of gravid female sand flies to substrates containing second/third-instar larvae (conditioned) and substrates aged the same amount of time without larvae (aged).

**Graphical Abstract:**

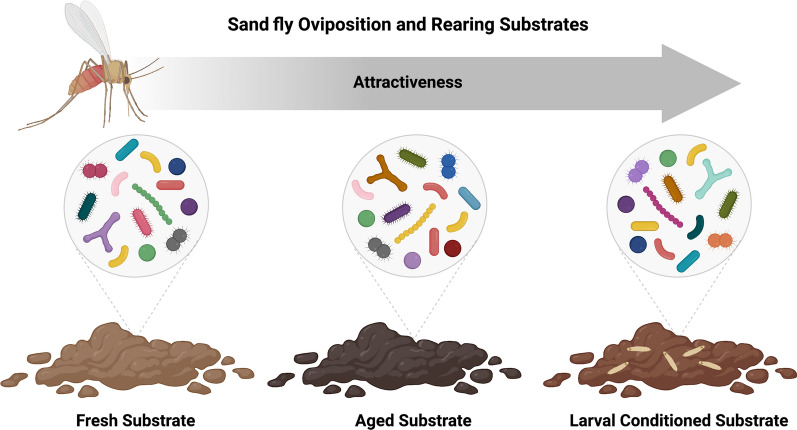

**Supplementary Information:**

The online version contains supplementary material available at 10.1186/s13071-022-05381-w.

## Background

Phlebotomine sand flies (Diptera: Psychodidae) are insect vectors that transmit protozoan parasites, as well as bacterial pathogens and viruses [1, 2]. Among the sand fly-transmitted diseases, leishmaniases, caused by obligate intracellular protozoa of the genus *Leishmania*, are the most significant. Leishmaniases are considered neglected tropical diseases due to their prevalence in regions of the world with a high index of poverty [3]. It is estimated that 350 million people are at risk of being infected by *Leishmania*, and there are 12 million new cases every year [4–6]. The lack of funding, reporting, good health care programs, and prevention systems make leishmaniases an even greater concern [7]. With no vaccine available, the use of personal protection (e.g., repellents, insecticide-treated clothing, or bed-nets) and residual spraying with insecticides to reduce exposure to sand fly bites currently represent the main means of leishmaniasis prevention [8, 9]. However, residual insecticide sprays also affect a wide range of non-target insects, and their efficacy is decreasing due to the evolution of insecticide resistance [8, 9]. Hence, a more targeted and efficient control method is urgently needed [10]. One promising control strategy is the attract-and-kill approach, where the main goal is to lure the vector to an insecticide using attractants. In particular, oviposition attractants are expected to be highly effective since they reduce pathogen transmission and control population growth by targeting older females that have blood-fed at least once and are thus more likely to be infected with pathogens [11–13]. For example, a lethal oviposition trap using a bait that consists of five attractive microbes isolated from leaf litter [14], substantially reduced mosquito (*Aedes aegypti*) abundance in Peru and Thailand [13]. Unfortunately, such a trap does not yet exist for sand flies.

Sand flies, unlike most biting *Diptera*, have a fully terrestrial life-cycle [1, 2]. Eggs are typically laid in soil rich in organic material on which the larvae feed and develop through four instars before pupation and adult emergence [15]. The coprophagic diet of larvae is consistent with the strong preference of New and Old World sand flies for different sources of decomposing organic matter, often from fecal sources [16–19], and there is strong evidence that microbes play an important role in mediating this attraction [20, 21]. There is also some evidence that bacteria are beneficial for the development of larvae [20], but this concept is still poorly understood.

The presence of early sand fly life stages (e.g., eggs, first-instar larvae) in rearing substrates has been shown to be attractive to gravid sand fly females [22, 23]. Furthermore, it was demonstrated that the presence of feeding and defecating larvae renders rearing substrates (herein termed “conditioned substrates”) more attractive and stimulatory to *Phlebotomus papatasi* sand flies than fresh larval rearing media or old (expired) rearing media from which all pupae have eclosed [19]. However, it was not clear whether the increased attraction was caused by larval conditioning, aging of the media, or a combination of both. To evaluate this possibility, Faw et al. [24] tested the effect of larval substrate conditioning on attraction and oviposition responses of *Ph. papatasi* sand flies while controlling for the effect of substrate aging. This study verified that gravid females were more attracted to conditioned substrates than to unconditioned substrates aged for the same amount of time (herein termed “aged substrates”) [24]. However, attraction and oviposition responses increased significantly for both larval conditioned and aged substrates when compared with the initial fresh larval food (herein termed “fresh substrates”) [24].

Given that attraction to decomposing organic matter is often mediated by bacterially produced semiochemicals [14, 20], the results of Faw et al. [24] suggested that conditioning and aging of rearing substrates may induce changes in the microbial community structure, thereby affecting their attractiveness to gravid sand fly females. Consistent with this hypothesis was the finding that some bacteria isolated from larval conditioned substrates [19] were highly attractive to gravid *Ph. papatasi* females [21]. Specifically, three individual isolates (*Microbacterium sorbitolivorans*, *Bacillus zhangzhouensis*, *Sphingobacterium phlebotomi*) were particularly attractive at low doses. Similarly, a mixture of 12 bacterial isolates was also attractive at low doses but at high doses was highly repellant [21]. Although informative, this study [21] was based on only a few culturable isolates and therefore, did not represent the entire bacterial community of the substrates. Furthermore, the bacterial community structure among conditioned and aged substrates or the changes within each over time has not been compared.

Here, we used 16S rRNA amplicon sequencing to (i) test the effect of larval substrate conditioning on bacterial community structure and (ii) characterize and compare the bacterial community composition of conditioned and aged substrates across the life-cycle stages of developing sand flies. We hypothesized that following larval introduction, the bacterial community structure of conditioned and aged substrates would diverge (i.e., become less similar). However, after all pupae had eclosed, we expected the bacterial communities of both substrate types to decrease in diversity and become more similar. Overall, our results were consistent with these predictions. Furthermore, we identified some bacteria that might be responsible for attraction to conditioned and aged substrates, which could be potential candidates for the development of oviposition lures that can be used for the control and surveillance of sand flies.

## Methods

### Sand fly colony maintenance

Sand flies (*Ph. papatasi*) from Abkük, Turkey (2004), were maintained at the Ecology of Infectious Disease Laboratory at the University of North Carolina at Greensboro (UNCG). The sand flies were reared using the mass-rearing methods described in [25] and maintained in incubators at 26 °C, 80% relative humidity (RH), and 14:10 light/dark photoperiod cycle. Female flies were blood-fed on live anesthetized mice (Harlan) (UNCG protocol # 20-0011, June 2020). Adults were fed with a 30% sucrose solution. Larvae were maintained in Nalgene jars with a 2.2 cm layer of plaster of Paris on the bottom and fed with fresh larval food which is a mixture of rabbit feces and rabbit chow in a 1:1 ratio.

### Experimental design

To characterize the bacterial communities of the conditioned and aged larval rearing substrates, five batches of fresh larval substrates were used as the starting point (baseline) of the experiment. This source was sampled (week 0; *n* = 5) and then utilized to produce two types of experimental substrates: larval conditioned substrates and aged non-larval conditioned substrates. Each substrate had five replicates. To start the experiment,  ~ 2500 surface-sterilized (1% bleach) eggs were placed in each of the five conditioned replicates. No eggs were placed in the five replicate aged substrates. Both substrate types were kept under the same conditions. Samples from conditioned (larva/pupa were removed from the substrate samples) and aged substrates were collected at week 2 (*n* = 10), week 4 (*n* = 10), and week 6 (*n* = 10), which spans the entire larval developmental period to adulthood. In the ninth week, one last group of samples were collected (*n* = 10) to obtain expired (where all pupae have matured and eclosed) substrate samples. In total, 45 samples were collected.

### DNA extraction and sequencing

DNA of the samples collected from the experiment was extracted using the PureLink™ Microbiome DNA Purification Kit (Invitrogen™). Extracted DNA was then used to perform a two-step 16S ribosomal RNA (rRNA) gene (V4 region) library preparation [26]. For the first step, polymerase chain reaction (PCR) amplification was performed using the primers 515F and 806R with Illumina platform-specific sequence adaptors attached: Hyb515F: 5′-TCGTCGGCAGCGTCAGATGTGTATAAGAGACAG**GTGYCAGCMGCCGCGGTA**-3′ and Hyb806R: 5′-GTCTCGTGGGCTCGGAGATGTGTATAAGAGACAG**GGACTACHVGGGTWTCTAAT**-3′. PCR cycling conditions were 98 °C for 30 s followed by 20 cycles of 98 °C (10 s), 58 °C (30 s), and 72 °C (30 s), with a final extension at 72 °C for 7 m. The resulting PCR product was cleaned using an Axygen™ AxyPrep Mag™ PCR Clean-up Kit. For the second step, the amplicons were indexed using the Illumina Nextera XT Index Kit v2 set D. PCR cycling conditions were 98 °C for 2 m followed by 15 cycles of 98 °C (10 s), 55 °C (30 s), 72 °C (30 s), with a final extension at 72 °C for 7 m. The final indexed amplicons were cleaned using the Axygen™ AxyPrep Mag™ PCR Clean-up Kit. Final clean amplicons were quantified with a Qubit 3.0 fluorometer (Life Technologies) with the dsDNA [double-stranded DNA] BR Assay Kit and pooled in equal concentrations for sequencing. A PhiX spike-in of 30% was added to the pooled library before sequencing to increase diversity on the run. The 16S amplicon sequencing was performed in-house on an Illumina iSeq 100 with 2 × 150 paired-end reads.

### Sequence analysis

The total number of reads passing the filter obtained from the sequencing run was 5,009,468. Forward and reverse reads were merged using Fast Length Adjustment of SHort reads (FLASH) [27] with minimum overlap of five base pairs (bp). Joined reads were quality-filtered in Qiime2 [28] using the DADA2 (divisive amplicon denoising algorithm) [29] pipeline, which includes removal of PhiX and chimeric reads. The data were then filtered to remove all sequences corresponding to mitochondria, chloroplast, and unassigned taxa. Further filtering was performed to remove any amplicon sequence variants (ASVs) that were represented by fewer than 10 reads. After quality filtering, we obtained 1,367,011 reads with a mean frequency of 30,512 reads per sample and 530 ASVs. The negative control contained only 19 reads that represented only a single ASV that was not detected in any other samples. Downstream analyses were performed in Qiime2 [28] at a sampling depth of 4000 reads per sample. This sampling depth was chosen so that all samples (aside from the negative control which only had 19 reads) could be included in the analysis while still maintaining enough reads per sample to capture the richness of the dataset.

To perform a phylogenetic diversity analysis, a tree was created using the script “qiime phylogeny align-to-tree-mafft-fasttree” [30, 31] Alpha and beta diversity analyses were then conducted using the script “qiime diversity core-metrics-phylogenetic” [28]. Alpha and beta diversity group significance was tested using the scripts “qiime diversity alpha-group-significance” and “qiime diversity beta-group-significance” [28]. Beta diversity was analyzed using two different methods, Bray–Curtis dissimilarity and weighted UniFrac [32] taxonomic assignment was conducted using the script “qiime feature-classifier classify-sklearn” [33] using a classifier trained on the Silva 16S (release 138) reference database [34] and based on the specific primers that will be used for amplification and the length of the sequence reads. For presence/absence, relative abundance, and differential abundance analyses, ASVs were evaluated at the “species” level (i.e., level-7 in qiime2). For details on individual sample information, including relative abundance of ASVs and level-7 taxa, see Additional file 1: Dataset S1.

### Statistical analysis and data visualization

Statistical analyses of alpha diversity were conducted in Qiime2 [28] using the Kruskal–Wallis test. Alpha diversity results generated in Qiime2 [28] were plotted in R [35]. Statistical analyses of beta diversity were conducted in Qiime2 using the permutational multivariate analysis of variance (PERMANOVA) test with 999 permutations. Principal coordinate analysis (PCoA) plots of beta diversity with 95% confidence intervals (stat_ellipse) were generated using Qiime2R [36]. The differential abundance of taxa was tested using analysis of compositions of microbiomes (ANCOM) [37] implemented in Qiime2. Pseudocounts were added to the data using “qiime composition add-pseudocount” before running ANCOM to remove zeros. ANCOM differential abundance volcano plots were generated in R [35].

## Results

### Alpha diversity patterns in aged and conditioned substrates

**Fig. 1:**
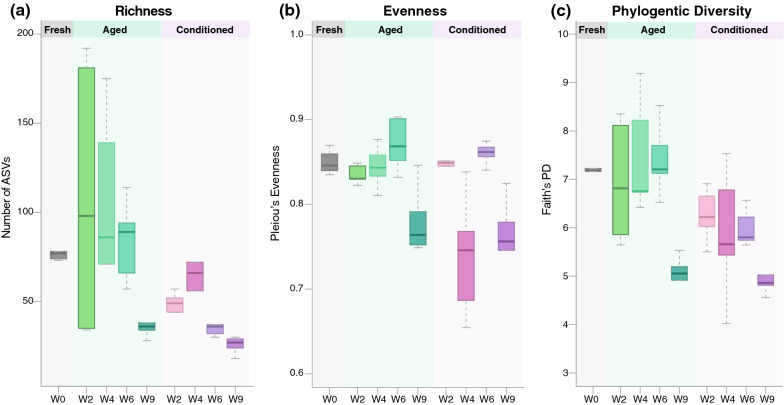
Alpha diversity measures of fresh, aged, and conditioned substrates at each sampling time point. Boxplots represent **a** richness with reference to the observed number of ASVs, **b** evenness based on Pielou’s evenness index, and **c** phylogenetic diversity measured by Faith’s phylogenetic diversity (PD) index. All test statistic values based on the Kruskal–Wallis test are listed in Additional file 2: Tables S1–S3

After quality filtering, a total of 530 bacterial amplicon sequence variants (ASVs) were identified. No archaeal ASVs were detected in any samples (Additional file 1: Dataset S1). Overall, more ASVs were present in aged substrates than in fresh or conditioned substrates (Fig. 1a). We observed a general trend of decreasing ASV richness over time in both aged and conditioned substrates (Fig. 1a). For the aged substrates, week 9 substrates had significantly lower richness than week 4 (*H* = 6.860, *df* = 8, *P* = 0.009) and week 6 substrates (*H* = 6.818, *df* = 8, *P* = 0.009). Within the conditioned substrates, week 9 was significantly lower in richness than all other weeks (*H* = 6.818, *df* = 8, *P* = 0.009). Considering the aged and conditioned substrates at the same time points, significant differences were found at week 6 (*H* = 6.818, *df* = 8, *P* = 0.009) and week 9 (*H* = 4.811, *df* = 8, *P* = 0.028), with higher richness in the aged substrates (Fig. 1a). Compared to the baseline (fresh substrates), the aged substrates were only found to be significantly different at week 9 (*H* = 6.818, *df* = 8, *P* = 0.009). In contrast, the richness of the conditioned substrates was significantly lower than that of the fresh substrates at all time points except for week 4 (see Additional file 2: Table S1 for all test statistic values).

In terms of evenness, the bacterial communities of the aged substrates were overall more even than those of the conditioned substrates (Fig. 1b). However, this difference was only significant at week 4 (*H* = 4.811, *df* = 8, *P* = 0.028). Within the aged substrates, evenness was similar during the first three time points and did not differ from that of the fresh substrates, but then dropped significantly at week 9 (Fig. 1b; see Additional file 2: Table S2 for all test statistic values). Regarding the conditioned substrates, evenness varied substantially, with no clear trend over time (Fig. 1b), with evenness at week 4 (*H* = 5.771, *df* = 8, *P* = 0.016) and week 9 (*H* = 6.818, *df* = 8, *P* = 0.009) being significantly lower than that of the fresh substrates.

Consistent with the richness and evenness indices, phylogenetic diversity was higher in aged substrates than in conditioned substrates (Fig. 1c; see Additional file 2: Table S3 for all test statistic values). Within the aged and conditioned substrates, phylogenetic diversity was similar across the first three time points but then decreased significantly in both groups at week 9 (Fig. 1c; Additional file 2: Table S3). At the first three time points, the phylogenetic diversity of the aged substrates did not differ significantly from that of the fresh substrates, but did at week 9 (*H* = 6.818, *df* = 8, *P* = 0.009). The conditioned substrates followed the same trend, with only week 9 substrates (*H* = 6.818, *df* = 8, *P* = 0.009) being significantly different from the fresh substrates (Fig. 1c).

### Comparison of bacterial community composition between aged and conditioned substrates

**Fig. 2:**
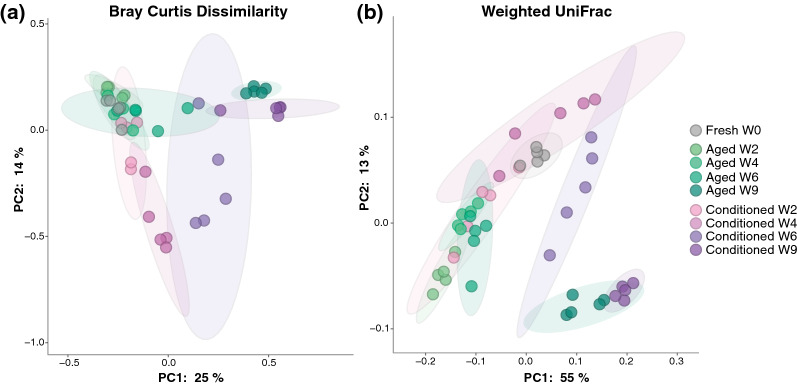
Beta diversity comparisons of fresh media, aged substrates, and conditioned substrates at each sampling time point. Principal coordinate analysis (PCoA) based on **a** Bray–Curtis dissimilarity, **b** weighted UniFrac. Significance was tested using PERMANOVA with 999 permutations. All test statistic values are listed in Additional file 2: Tables S4, S5

Overall, we found that bacterial community compositions differed between aged and conditioned substrates as well as temporally within each substrate. Based on the quantitative non-phylogenetic beta diversity metric, Bray–Curtis dissimilarity (Fig. 2a), bacterial community composition of fresh substrates significantly differed from that of the conditioned and aged substrates at all time points (see Additional file 2: Table S4 for all test statistic values). Significant differences were also found between conditioned and aged substrates at week 2 (pseudo-*F* = 1.697, *P* = 0.018), week 4 (pseudo-*F* = 5.331, *P* = 0.006), week 6 (pseudo-*F* = 3.649, *P* = 0.013), and week 9 (pseudo-*F* = 2.285, *P* = 0.008). When looking at the differences in community composition within each substrate type, the conditioned substrates differed significantly from each other at every time point (Additional file 2: Table S4). For the aged substrates, the bacterial communities at each time point, except for week 4 versus week 6 (pseudo-*F* = 1.167, *P* = 0.196), were also significantly different (Additional file 2: Table S4). Consistent results were found based on weighted UniFrac analysis (a quantitative method that incorporates phylogenetic distances), with the only differences being that week 4 and week 6 conditioned substrates were not found to be significantly different (pseudo-*F* = 2.585, *P* = 0.073) and aged week 4 and week 6 substrates differed significantly (pseudo-*F* = 2.011, *P* = 0.009) (Fig. 2b; see Additional file 2: Table S5 for all test statistic values).

### Characterization of the bacterial communities from fresh, aged, and conditioned substrates

**Fig. 3:**
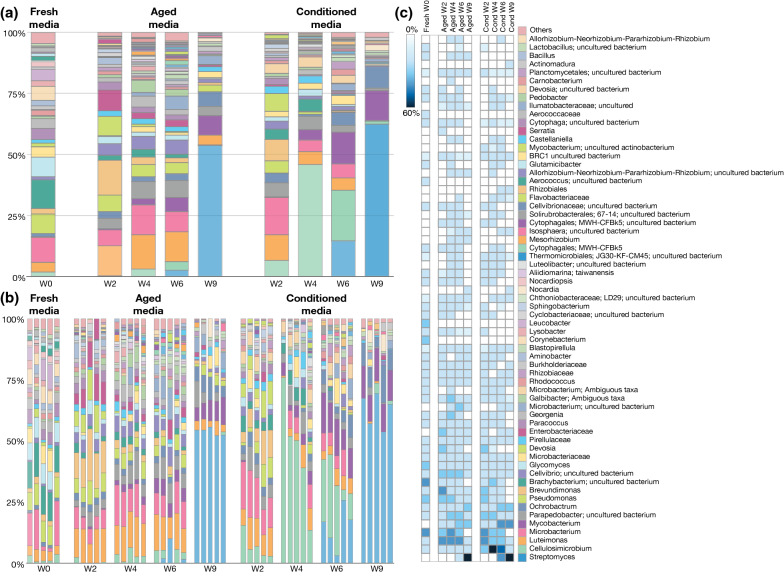
Relative abundance of taxa in fresh, aged, and conditioned substrates and each sampling time point. **a** Average relative abundance of the bacterial communities of fresh larval media (week 0) and aged and conditioned substrates at weeks 2, 4, 6, and 9. **b** Bacterial relative abundance in individual replicates of fresh larval media (week 0) and aged and conditioned substrates at different time points (weeks 2, 4, 6, and 9). Each bar represents an individual sample. Colors represent the relative abundance of each taxon. **c** Relative abundance heatmap of taxa found in fresh, aged, and conditioned substrates at each time point. Taxa present in less than 1% total relative abundance were combined and are listed as “others.” See Additional file 1: Dataset S1 for full relative abundance data

The 530 unique ASVs identified across all samples corresponded to 116 different taxa based on “species” level taxonomic clustering (Fig. 3, Additional file 1: Dataset S1). As indicated by the beta diversity analysis, we observed clear differences in community composition across all substrate types and time points (Fig. 3a, b). Particularly, *Cellulosimicrobium* was present in much higher relative abundance in conditioned week 4 and week 6 substrates than in any other substrate. Additionally, *Streptomyces* made up over 50% of the community in both aged and conditioned substrates at week 9 (Fig. 3a, b).

To visualize the differential abundance of taxa more easily, the average relative abundance of each taxon across groups at each time point was plotted using a heatmap (Fig. 3c). Three taxa were found only in fresh substrates (*Corynebacterium*, *Leucobacter*, *Aerococcus*), and 20 taxa were present in aged and/or conditioned that were not identified in fresh substrates (Fig. 3c). One taxon was unique to aged week 2 (*Serratia*), one to aged week 4 (*Carnobacterium*), and one to conditioned week 9 (*Actinomadura*). Additionally, only one taxon was unique to a particular time point (i.e., *Nocardia* was only present in week 9 aged and conditioned substrates).

**Fig. 4:**
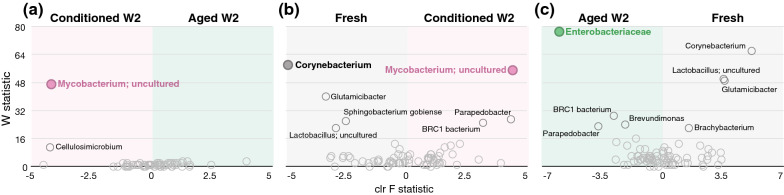
Analysis of composition of microbiomes (ANCOM) differential abundance volcano plots. The *y*-axis represents the W statistic value or the number of times the null hypothesis was rejected. The *x*-axis value represents the centered log ratio (clr) transformed F statistic (the effect size difference for a particular taxon between groups). Taxa with reject null hypothesis are shown in filled circles (green, pink, or gray) and labeled with taxon name. Taxa that were not significant but displayed a higher-than-average W value are also labeled with taxon name. **a** Conditioned versus aged week 2 substrates, **b** fresh versus conditioned week 2 substrates, and **c** fresh versus aged week 2 substrates

Because previous studies have demonstrated that female sand flies are more attracted to week-2 conditioned (second/third-instar larvae) and week-2 aged substrates (19, 24) than to fresh substrates, we specifically compared the differential abundance of taxa between these groups, with the goal of identifying bacteria that could potentially be responsible for attraction/repulsion. When comparing conditioned and aged substrates at week 2, *Mycobacterium* (uncultured actinobacterium) was significantly more abundant in conditioned than in aged substrates (Fig. 4a), and in fact, was never observed in the fresh or aged substrates at any time point (Additional file 1: Dataset S1). *Cellulosimicrobium* was also present in higher relative abundance in conditioned week 2 than aged week-2 substrates; however, this difference was not statistically significant (Fig. 4a). *Corynebacterium* was only found in fresh substrates (Fig. 4b, c, Additional file 1: Dataset S1). Several other taxa were more abundant in fresh (*Glutamicibacter*, *Sphingobacterium gobiense*, and *Lactobacillus*) or conditioned week 2 substrates (*Parapedobacter* and BRC1 bacterium), but not significantly so (Fig. 4b). *Enterobacteriaceae* was significantly more abundant in aged week-2 substrates than in fresh substrates and was not observed in any of the fresh substrate samples (Fig. 4c, Additional file 1: Dataset S1). Some other taxa were present in higher abundance in aged (*Parapedobacter*, BRC1 bacterium, and *Brevundimonas*) or in fresh (*Corynebacterium*, *Lactobacillus*, *Glutamicibacter*, *Brachybacterium*) substrates, but were not found to be statistically significant (Fig. 4c).

Based on presence–absence patterns (Additional file 1: Dataset S1), five other taxa, not identified as significant in the ANCOM [37] analysis, were observed in conditioned week-2 but not aged week 2 substrates (*Microbacterium* ambiguous taxa, *Moheibacter* uncultured bacterium, Thermomicrobiales JG30-KF-CM45, *Lactobacillus* uncultured bacterium, and *Luteolibacter* uncultured bacterium), and a total of 16 taxa were observed in week 2 aged but not in week 2 conditioned substrates (*Iamia*, Ilumatobacteraceae, Cytophagales MWH-CFBk5, *Myroides*, *Pedobacter*, *Enterococcus*, *Devosia*, Chthoniobacteraceae LD29, *Luteimonas*, *Pseudofulvimonas*, *Steroidobacter*, *Pseudohongiella*, *Serratia*, *Pelagibacterium*, *Chloroflexi* bacterium, and *Sphingobacterium gobiense*). Four taxa were present in week 2 aged and conditioned substrates but absent in fresh substrates (*Cytophagales* MWH-CFBk5 uncultured bacterium, *Shinella*, *Enterobacteriaceae*, and a *Mycobacterium* sp.).

## Discussion

Our study demonstrates that the presence of sand fly larvae causes major shifts in the bacterial community composition of larval sand fly rearing substrates. Rearing substrates devoid of larvae but aged for the same amount of time also underwent changes in bacterial community composition, resulting in significant differences between aged and conditioned substrates. These results are reflected in the observed patterns of bacterial diversity, community similarity, and taxa composition across substrate types and time points. In general, alpha diversity (species richness, evenness, and phylogenetic diversity) of the aged substrates was more similar to the baseline fresh substrates than to the conditioned substrates. In the conditioned substrates, all diversity indices tended to be lower than the fresh substrates and the aged substrates. However, at week 9, both aged and conditioned substrates significantly decreased in richness, evenness, and phylogenetic diversity and converged to similar values. Complementary to the alpha-diversity patterns, we observed differences in bacterial community composition (beta diversity) between all substrate types, with the communities becoming less similar over time but then starting to converge at week 9. Taken together, our results indicate that aged and conditioned substrates differ in community diversity and structure and that the presence of developing larvae reduces the bacterial diversity of the rearing substrates more quickly than the aging process alone.

Resource quantity and quality are well known to affect biodiversity in ecological systems [26]. Here, we found that bacterial community diversity in the conditioned substrates substantially decreased soon after the introduction of larvae (i.e., week 2), whereas diversity in the aged substrates did not decrease until week 9. The decrease in bacterial diversity in substrates containing developing larvae (conditioned substrates), could be the result of competition between the larvae and bacteria for essential nutrients or even the direct consumption of the bacteria by the larvae. It is also possible that larval excreta contain noxious compounds that decrease the quality of the substrate or are toxic to some bacteria. Consistent with these hypotheses, we found that after the larvae pupate (week 6), the bacterial community composition of the conditioned substrates started to converge and became more similar to the aged substrates. Thus, our findings suggest that once larvae are no longer present, the larval-conditioned substrate starts going through the standard process of decomposition, resulting in the bacterial community becoming more similar to that of the aged substrate.

The observed changes in bacterial diversity could be associated with the previously reported increase in the attractiveness of conditioned substrates [19, 24]. It is possible that larval conditioning leads to a decrease in the relative abundance of bacteria that are unattractive or even repellent and/or to an increase in the relative abundance of bacteria that are attractive to sand flies. In a previous study, Kakumanu et al. [21] identified three bacterial isolates (*Microbacterium sorbitolivorans*, *Bacillus zhangzhouensis*, *Sphingobacterium phlebotomi*) that were highly attractive to sand flies at low concentrations but were repellant at high concentrations. In contrast, *Sphingobacterium daejeonense* was repellent at low concentration but tended to be attractive (but not significantly so) at high concentrations. Similarly, *Leucobacter holotrichiae* and *Pseudomonas nitrititolerans* were repellent at low but attractive at high concentrations, while *Alcaligenes faecalis* was repellent but only at low concentrations [21]. Bacteria from all these genera were found in our analyses. However, given that our resolution was limited to the genus level (or above), we cannot confirm the presence or absence of these specific bacterial species in our dataset. Given our ability to characterize the entire bacterial community, albeit at low resolution, we looked for taxa that could be associated with the attraction or repellence of female sand flies by comparing fresh, conditioned week 2 and aged week 2 substrates, which correspond to the substrates previously studied for attraction [24]. We found several taxa that were unique to either fresh substrates, week 2 conditioned substrates, or week 2 aged substrates (see “Results” section). We also identified some taxa that were present in both aged and conditioned week 2 substrates but absent in fresh substrates (*Ilumatobacteriaceae* uncultured bacterium, *Serratia*, *Mycobacterium* uncultured bacterium, *Cytophagales* MWH-CFBK5 uncultured bacterium, *Luteolibacter* uncultured bacterium, *Enterobacteriaceae*, and *Mycobacterium*). We speculate that taxa only observed in aged and/or conditioned substrates were present in fresh substrates but at very low relative abundance below the level of detection with our sequencing depth. Some of the taxa we observed (*Pseudomonas*, *Leucobacter*, *Bacillus*, *Cellulosimicrobium*, *Brevundimonas*, *Luteimonas*, *Sphingobacterium*, and *Microbacterium*) are from the same genera as the isolates identified as attractive or repellent by Kakumanu et al. [21], but none were unique to conditioned or aged substrates in our study. All taxa we identified to be unique to aged or conditioned substrates have yet to be investigated. It is important to note that we did not test the sand flies’ preference for the specific substrates that were analyzed in this study (although they are composed of the same ingredients as used in previous behavioral tests). Hence, our inferences are correlational and based on the assumption that the previously observed attraction patterns [19, 24] would be consistent here as well. Overall, our results provide an excellent starting point for future studies aiming to identify candidates for attract-and-kill strategies.

One caveat of our study is that we only characterized bacteria and did not account for other microorganisms, such as yeasts and fungi, that are expected to inhabit these substrates. Also, 16S rRNA amplicon sequencing can only reliably provide genus-level data (at best), which prevented us from characterizing the bacterial community at the species level; limiting our ability to accurately classify the identified taxa and compare them to other studies. Moreover, the available 16S reference databases are not complete, so many taxa could not even be classified to the genus level. In addition, it is also possible that the behavioral oviposition responses observed in previous studies [19, 24] may not necessarily be due to the presence, absence, or abundance of specific bacteria, but rather the result of bacterial community shifts and/or differences in the volatile compound profile of the substrate headspace. The idea of a microbiome-driven effect on insect oviposition-site selection (or any bacterially affected behavioral choice for that matter) is relatively novel and requires further investigation to assess its generality [38]. Practically, the implication of an entire community being responsible for attractiveness is quite disconcerting as the re-creation of such a diverse and complex community would be challenging. In such a case, studying and replicating the volatile composition of the headspace may provide an alternative solution. We would also like to note that prior to starting our experiments, we surface-sterilized the eggs. We acknowledge that it is possible that microbes on the surface of the eggs could impact the community composition of the substrate. However, since it is unclear if and what microbes are present on sand fly eggs, we sterilized them to ensure we were testing only the effect of larval conditioning and to limit differences across replicates. Future studies are needed to determine whether sand fly eggs contain microbes and if they contribute to larval development and/or substrate composition.

## Conclusion

This study demonstrated a unique phenomenon in which preimaginal stages of sand flies induce a shift in the bacterial community structure of their rearing substrate, which we hypothesize is responsible for enhancing the attraction of gravid female sand flies to that substrate. Our findings, along with others, indicate that preimaginal conspecific stages may have both a direct [23] and an indirect [19] effect on the oviposition site selection choices of gravid females. The reason for such a preference is not yet understood but is assumed to be correlated with oviposition site quality [20]. By temporally characterizing the bacterial composition of fresh, aged, and larval-conditioned substrates, we have provided, for the first time, clear evidence that substrate decomposition (aging) and larval conditioning alters the bacterial community of sand fly rearing substrates, which in turn may affect the oviposition site selection behavior of gravid sand flies.

## Supplementary Information

**Additional file 1: Dataset S1.** Number of reads for each ASV (tab 1) and percent relative abundance of ASVs clustered to level-7 taxonomy, e.g., “species” (tab 2).

**Additional file 2: Table S1.** Pairwise comparisons between substrate types at each time point based on the number of ASVs (richness). All test statistic values were calculated with the Kruskal–Wallis test. **Table S2.** Pairwise comparisons between substrate types at each time point based on Pielou’s evenness index. All test statistic values were calculated with the Kruskal–Wallis test. **Table S3.** Pairwise comparisons between substrate types at each time point based on Faith’s Phylogenetic Diversity (PD) index. All test statistic values were calculated with the Kruskal–Wallis test. **Table S4.** Pairwise comparisons between substrate types at each time point based on the Bray–Curtis dissimilarity index. All test statistic values were calculated with the PERMANOVA test with 999 permutations. **Table S5.** Pairwise comparisons between substrate types at each time point based on the weighted UniFrac index. All test statistic values were calculated with the PERMANOVA test with 999 permutations.

## Data Availability

The datasets supporting the conclusions of this article are available in the Sequence Read Archive (SRA) at NCBI under BioProject PRJNA806848. All other data generated or analyzed during this study are included in this published article [and its additional information files].

## Abbreviations

ASV: Amplicon sequence variant
RH: Relative humidity
PCR: Polymerase chain reaction
*Ph*: *Phlebotomus*
ANCOM: Analysis of compositions of microbiomes
Qiime: Quantitative insights into microbial ecology
FLASH: Fast Length Adjustment of SHort reads
DADA: Divisive amplicon denoising algorithm
PERMANOVA: Permutational multivariate analysis of variance
