# Supplementary material for: Microbial ecology of sand fly breeding sites: aging and larval conditioning alter the bacterial community composition of rearing substrates
Source: Parasit Vectors. 2022 Jul 26;15:265. doi: 10.1186/s13071-022-05381-w (PMC9327230; doi:10.1186/s13071-022-05381-w)
Supplement: Supplementary file 2 — Additional file 2: Table S1. Pairwise comparisons between substrate types at each time point based on the number of ASVs (richness). All test statistic values were calculated with the Kruskal–Wallis test. Table S2. Pairwise comparisons between substrate types at each time point based on Pielou’s evenness index. All test statistic values were calculated with the Kruskal–Wallis test. Table S3. Pairwise comparisons between substrate types at each time point based on Faith’s Phylogenetic Diversity (PD) index. All test statistic values were calculated with the Kruskal–Wallis test. Table S4. Pairwise comparisons between substrate types at each time point based on the Bray–Curtis dissimilarity index. All test statistic values were calculated with the PERMANOVA test with 999 permutations. Table S5. Pairwise comparisons between substrate types at each time point based on the weighted UniFrac index. All test statistic values were calculated with the PERMANOVA test with 999 permutations. [file 13071_2022_5381_MOESM2_ESM.pdf]

Supplementary Tables

Group 1	Group 2	H	p-value	q-value
**Aged_week2 (n=5)**	Aged_week4 (n=5)	0.011	0.917	0.917
**Aged_week2 (n=5)**	Aged_week6 (n=5)	0.098	0.754	0.798
**Aged_week2 (n=5)**	Aged_week9 (n=5)	1.580	0.209	0.301
**Aged_week2 (n=5)**	Conditioned_week2 (n=5)	0.884	0.347	0.446
**Aged_week2 (n=5)**	Conditioned_week4 (n=5)	0.535	0.465	0.558
**Aged_week2 (n=5)**	Conditioned_week6 (n=5)	1.844	0.175	0.262
**Aged_week2 (n=5)**	Conditioned_week9 (n=5)	6.818	0.009	0.023
**Aged_week2 (n=5)**	Fresh_week0 (n=5)	0.273	0.602	0.656
**Aged_week4 (n=5)**	Aged_week6 (n=5)	0.538	0.463	0.558
**Aged_week4 (n=5)**	Aged_week9 (n=5)	6.860	0.009	0.023
**Aged_week4 (n=5)**	Conditioned_week2 (n=5)	6.860	0.009	0.023
**Aged_week4 (n=5)**	Conditioned_week4 (n=5)	2.470	0.116	0.192
**Aged_week4 (n=5)**	Conditioned_week6 (n=5)	6.860	0.009	0.023
**Aged_week4 (n=5)**	Conditioned_week9 (n=5)	6.860	0.009	0.023
**Aged_week4 (n=5)**	Fresh_week0 (n=5)	0.274	0.600	0.656
**Aged_week6 (n=5)**	Aged_week9 (n=5)	6.818	0.009	0.023
**Aged_week6 (n=5)**	Conditioned_week2 (n=5)	6.322	0.012	0.027
**Aged_week6 (n=5)**	Conditioned_week4 (n=5)	0.894	0.344	0.446
**Aged_week6 (n=5)**	Conditioned_week6 (n=5)	6.818	0.009	0.023
**Aged_week6 (n=5)**	Conditioned_week9 (n=5)	6.818	0.009	0.023
**Aged_week6 (n=5)**	Fresh_week0 (n=5)	0.273	0.602	0.656
**Aged_week9 (n=5)**	Conditioned_week2 (n=5)	1.320	0.251	0.347
**Aged_week9 (n=5)**	Conditioned_week4 (n=5)	3.153	0.076	0.144
**Aged_week9 (n=5)**	Conditioned_week6 (n=5)	0.044	0.834	0.858
**Aged_week9 (n=5)**	Conditioned_week9 (n=5)	4.811	0.028	0.060
**Aged_week9 (n=5)**	Fresh_week0 (n=5)	6.818	0.009	0.023
**Conditioned_week2 (n=5)**	Conditioned_week4 (n=5)	2.151	0.142	0.223
**Conditioned_week2 (n=5)**	Conditioned_week6 (n=5)	2.455	0.117	0.192
**Conditioned_week2 (n=5)**	Conditioned_week9 (n=5)	6.818	0.009	0.023
**Conditioned_week2 (n=5)**	Fresh_week0 (n=5)	6.818	0.009	0.023
**Conditioned_week4 (n=5)**	Conditioned_week6 (n=5)	3.153	0.076	0.144
**Conditioned_week4 (n=5)**	Conditioned_week9 (n=5)	6.818	0.009	0.023
**Conditioned_week4 (n=5)**	Fresh_week0 (n=5)	2.455	0.117	0.192
**Conditioned_week6 (n=5)**	Conditioned_week9 (n=5)	6.322	0.012	0.027
**Conditioned_week6 (n=5)**	Fresh_week0 (n=5)	6.818	0.009	0.023
**Conditioned_week9 (n=5)**	Fresh_week0 (n=5)	6.818	0.009	0.023

Table S1. Pairwise comparisons between substrate types at each timepoint based on the number of ASVs (richness). All p-values were calculated with the Kruskal Wallis test.

Group 1	Group 2	H	p-value	q-value
**Aged_week2 (n=5)**	Aged_week4 (n=5)	0.535	0.465	0.540
**Aged_week2 (n=5)**	Aged_week6 (n=5)	4.811	0.028	0.064
**Aged_week2 (n=5)**	Aged_week9 (n=5)	3.153	0.076	0.136
**Aged_week2 (n=5)**	Conditioned_week2 (n=5)	1.844	0.175	0.262
**Aged_week2 (n=5)**	Conditioned_week4 (n=5)	3.938	0.047	0.089
**Aged_week2 (n=5)**	Conditioned_week6 (n=5)	4.811	0.028	0.064
**Aged_week2 (n=5)**	Conditioned_week9 (n=5)	5.771	0.016	0.049
**Aged_week2 (n=5)**	Fresh_week0 (n=5)	2.455	0.117	0.192
**Aged_week4 (n=5)**	Aged_week6 (n=5)	1.320	0.251	0.334
**Aged_week4 (n=5)**	Aged_week9 (n=5)	3.938	0.047	0.089
**Aged_week4 (n=5)**	Conditioned_week2 (n=5)	0.098	0.754	0.776
**Aged_week4 (n=5)**	Conditioned_week4 (n=5)	4.811	0.028	0.064
**Aged_week4 (n=5)**	Conditioned_week6 (n=5)	0.884	0.347	0.431
**Aged_week4 (n=5)**	Conditioned_week9 (n=5)	5.771	0.016	0.049
**Aged_week4 (n=5)**	Fresh_week0 (n=5)	0.273	0.602	0.656
**Aged_week6 (n=5)**	Aged_week9 (n=5)	5.771	0.016	0.049
**Aged_week6 (n=5)**	Conditioned_week2 (n=5)	2.455	0.117	0.192
**Aged_week6 (n=5)**	Conditioned_week4 (n=5)	5.771	0.016	0.049
**Aged_week6 (n=5)**	Conditioned_week6 (n=5)	0.273	0.602	0.656
**Aged_week6 (n=5)**	Conditioned_week9 (n=5)	6.818	0.009	0.049
**Aged_week6 (n=5)**	Fresh_week0 (n=5)	0.884	0.347	0.431
**Aged_week9 (n=5)**	Conditioned_week2 (n=5)	4.811	0.028	0.064
**Aged_week9 (n=5)**	Conditioned_week4 (n=5)	1.320	0.251	0.334
**Aged_week9 (n=5)**	Conditioned_week6 (n=5)	5.771	0.016	0.049
**Aged_week9 (n=5)**	Conditioned_week9 (n=5)	0.535	0.465	0.540
**Aged_week9 (n=5)**	Fresh_week0 (n=5)	3.938	0.047	0.089
**Conditioned_week2 (n=5)**	Conditioned_week4 (n=5)	5.771	0.016	0.049
**Conditioned_week2 (n=5)**	Conditioned_week6 (n=5)	1.844	0.175	0.262
**Conditioned_week2 (n=5)**	Conditioned_week9 (n=5)	5.771	0.016	0.049
**Conditioned_week2 (n=5)**	Fresh_week0 (n=5)	0.011	0.917	0.917
**Conditioned_week4 (n=5)**	Conditioned_week6 (n=5)	6.818	0.009	0.049
**Conditioned_week4 (n=5)**	Conditioned_week9 (n=5)	0.098	0.754	0.776
**Conditioned_week4 (n=5)**	Fresh_week0 (n=5)	5.771	0.016	0.049
**Conditioned_week6 (n=5)**	Conditioned_week9 (n=5)	6.818	0.009	0.049
**Conditioned_week6 (n=5)**	Fresh_week0 (n=5)	1.320	0.251	0.334
**Conditioned_week9 (n=5)**	Fresh_week0 (n=5)	6.818	0.009	0.049

Table S2. Pairwise comparisons between substrate types at each timepoint based on Pielou's evenness index. All p-values were calculated with the Kruskal Wallis test.

Group 1	Group 2	H	p-value	q-value
**Aged_week2 (n=5)**	Aged_week4 (n=5)	0.273	0.602	0.699
**Aged_week2 (n=5)**	Aged_week6 (n=5)	0.535	0.465	0.577
**Aged_week2 (n=5)**	Aged_week9 (n=5)	6.818	0.009	0.030
**Aged_week2 (n=5)**	Conditioned_week2 (n=5)	0.535	0.465	0.577
**Aged_week2 (n=5)**	Conditioned_week4 (n=5)	1.844	0.175	0.251
**Aged_week2 (n=5)**	Conditioned_week6 (n=5)	1.844	0.175	0.251
**Aged_week2 (n=5)**	Conditioned_week9 (n=5)	6.818	0.009	0.030
**Aged_week2 (n=5)**	Fresh_week0 (n=5)	0.098	0.754	0.798
**Aged_week4 (n=5)**	Aged_week6 (n=5)	0.098	0.754	0.798
**Aged_week4 (n=5)**	Aged_week9 (n=5)	6.818	0.009	0.030
**Aged_week4 (n=5)**	Conditioned_week2 (n=5)	3.153	0.076	0.144
**Aged_week4 (n=5)**	Conditioned_week4 (n=5)	1.844	0.175	0.251
**Aged_week4 (n=5)**	Conditioned_week6 (n=5)	5.771	0.016	0.039
**Aged_week4 (n=5)**	Conditioned_week9 (n=5)	6.818	0.009	0.030
**Aged_week4 (n=5)**	Fresh_week0 (n=5)	0.011	0.917	0.917
**Aged_week6 (n=5)**	Aged_week9 (n=5)	6.818	0.009	0.030
**Aged_week6 (n=5)**	Conditioned_week2 (n=5)	4.811	0.028	0.060
**Aged_week6 (n=5)**	Conditioned_week4 (n=5)	3.153	0.076	0.144
**Aged_week6 (n=5)**	Conditioned_week6 (n=5)	5.771	0.016	0.039
**Aged_week6 (n=5)**	Conditioned_week9 (n=5)	6.818	0.009	0.030
**Aged_week6 (n=5)**	Fresh_week0 (n=5)	0.011	0.917	0.917
**Aged_week9 (n=5)**	Conditioned_week2 (n=5)	5.771	0.016	0.039
**Aged_week9 (n=5)**	Conditioned_week4 (n=5)	1.844	0.175	0.251
**Aged_week9 (n=5)**	Conditioned_week6 (n=5)	6.818	0.009	0.030
**Aged_week9 (n=5)**	Conditioned_week9 (n=5)	0.535	0.465	0.577
**Aged_week9 (n=5)**	Fresh_week0 (n=5)	6.818	0.009	0.030
**Conditioned_week2 (n=5)**	Conditioned_week4 (n=5)	0.273	0.602	0.699
**Conditioned_week2 (n=5)**	Conditioned_week6 (n=5)	0.884	0.347	0.481
**Conditioned_week2 (n=5)**	Conditioned_week9 (n=5)	6.818	0.009	0.030
**Conditioned_week2 (n=5)**	Fresh_week0 (n=5)	4.811	0.028	0.060
**Conditioned_week4 (n=5)**	Conditioned_week6 (n=5)	0.098	0.754	0.798
**Conditioned_week4 (n=5)**	Conditioned_week9 (n=5)	1.844	0.175	0.251
**Conditioned_week4 (n=5)**	Fresh_week0 (n=5)	2.455	0.117	0.211
**Conditioned_week6 (n=5)**	Conditioned_week9 (n=5)	6.818	0.009	0.030
**Conditioned_week6 (n=5)**	Fresh_week0 (n=5)	5.771	0.016	0.039
**Conditioned_week9 (n=5)**	Fresh_week0 (n=5)	6.818	0.009	0.030

Table S3. Pairwise comparisons between substrate types at each timepoint based on Faith's Phylogenetic Diversity (PD) index. All p-values were calculated with the Kruskal Wallis test.

Group 1	Group 2	Sample size	Permutations	pseudo-F	p-value	q-value
**Aged_week2**	Aged_week4	10	999	2.23	0.01	0.02
**Aged_week2**	Aged_week6	10	999	2.42	0.01	0.02
**Aged_week2**	Aged_week9	10	999	9.42	0.01	0.02
**Aged_week2**	Conditioned_week2	10	999	1.70	0.02	0.02
**Aged_week2**	Conditioned_week4	10	999	6.64	0.01	0.02
**Aged_week2**	Conditioned_week6	10	999	6.77	0.01	0.02
**Aged_week2**	Conditioned_week9	10	999	10.08	0.01	0.02
**Aged_week2**	Fresh_week0	10	999	4.23	0.01	0.02
**Aged_week4**	Aged_week6	10	999	1.17	0.20	0.20
**Aged_week4**	Aged_week9	10	999	9.46	0.01	0.02
**Aged_week4**	Conditioned_week2	10	999	2.18	0.02	0.02
**Aged_week4**	Conditioned_week4	10	999	5.33	0.01	0.02
**Aged_week4**	Conditioned_week6	10	999	5.78	0.01	0.02
**Aged_week4**	Conditioned_week9	10	999	9.88	0.02	0.02
**Aged_week4**	Fresh_week0	10	999	4.27	0.01	0.02
**Aged_week6**	Aged_week9	10	999	6.21	0.01	0.02
**Aged_week6**	Conditioned_week2	10	999	2.21	0.01	0.02
**Aged_week6**	Conditioned_week4	10	999	4.49	0.01	0.02
**Aged_week6**	Conditioned_week6	10	999	3.65	0.01	0.02
**Aged_week6**	Conditioned_week9	10	999	6.86	0.02	0.02
**Aged_week6**	Fresh_week0	10	999	4.26	0.01	0.02
**Aged_week9**	Conditioned_week2	10	999	6.84	0.01	0.02
**Aged_week9**	Conditioned_week4	10	999	11.40	0.01	0.02
**Aged_week9**	Conditioned_week6	10	999	6.61	0.01	0.02
**Aged_week9**	Conditioned_week9	10	999	2.28	0.01	0.02
**Aged_week9**	Fresh_week0	10	999	11.94	0.01	0.02
**Conditioned_week2**	Conditioned_week4	10	999	3.02	0.01	0.02
**Conditioned_week2**	Conditioned_week6	10	999	3.51	0.01	0.02
**Conditioned_week2**	Conditioned_week9	10	999	6.39	0.01	0.02
**Conditioned_week2**	Fresh_week0	10	999	3.11	0.01	0.02
**Conditioned_week4**	Conditioned_week6	10	999	3.34	0.02	0.02
**Conditioned_week4**	Conditioned_week9	10	999	9.73	0.01	0.02
**Conditioned_week4**	Fresh_week0	10	999	6.87	0.01	0.02
**Conditioned_week6**	Conditioned_week9	10	999	5.11	0.01	0.02
**Conditioned_week6**	Fresh_week0	10	999	8.27	0.01	0.02
**Conditioned_week9**	Fresh_week0	10	999	11.38	0.00	0.02

Table S4. Pairwise comparisons between substrate types at each timepoint based on the Bray Curtis Dissimilarity index. All p-values were calculated with the PERMANOVA test with 999 permutations.

Group 1	Group 2	Sample size	Permutations	pseudo-F	p-value	q-value
**Aged_week2**	Aged_week4	10	999	19.747	0.007	0.015
**Aged_week2**	Aged_week6	10	999	17.768	0.004	0.015
**Aged_week2**	Aged_week9	10	999	52.577	0.013	0.015
**Aged_week2**	Conditioned_week2	10	999	7.162	0.011	0.015
**Aged_week2**	Conditioned_week4	10	999	25.538	0.008	0.015
**Aged_week2**	Conditioned_week6	10	999	42.051	0.011	0.015
**Aged_week2**	Conditioned_week9	10	999	89.183	0.008	0.015
**Aged_week2**	Fresh_week0	10	999	63.129	0.007	0.015
**Aged_week4**	Aged_week6	10	999	2.011	0.009	0.015
**Aged_week4**	Aged_week9	10	999	34.945	0.010	0.015
**Aged_week4**	Conditioned_week2	10	999	5.285	0.008	0.015
**Aged_week4**	Conditioned_week4	10	999	13.053	0.013	0.015
**Aged_week4**	Conditioned_week6	10	999	22.602	0.011	0.015
**Aged_week4**	Conditioned_week9	10	999	66.043	0.013	0.015
**Aged_week4**	Fresh_week0	10	999	34.989	0.010	0.015
**Aged_week6**	Aged_week9	10	999	27.406	0.006	0.015
**Aged_week6**	Conditioned_week2	10	999	5.431	0.010	0.015
**Aged_week6**	Conditioned_week4	10	999	12.855	0.011	0.015
**Aged_week6**	Conditioned_week6	10	999	18.141	0.015	0.016
**Aged_week6**	Conditioned_week9	10	999	52.122	0.008	0.015
**Aged_week6**	Fresh_week0	10	999	33.346	0.014	0.016
**Aged_week9**	Conditioned_week2	10	999	18.033	0.011	0.015
**Aged_week9**	Conditioned_week4	10	999	7.565	0.005	0.015
**Aged_week9**	Conditioned_week6	10	999	6.837	0.010	0.015
**Aged_week9**	Conditioned_week9	10	999	5.278	0.007	0.015
**Aged_week9**	Fresh_week0	10	999	30.879	0.013	0.015
**Conditioned_week2**	Conditioned_week4	10	999	8.424	0.013	0.015
**Conditioned_week2**	Conditioned_week6	10	999	12.151	0.008	0.015
**Conditioned_week2**	Conditioned_week9	10	999	31.017	0.008	0.015
**Conditioned_week2**	Fresh_week0	10	999	13.224	0.008	0.015
**Conditioned_week4**	Conditioned_week6	10	999	2.585	0.073	0.073
**Conditioned_week4**	Conditioned_week9	10	999	9.363	0.010	0.015
**Conditioned_week4**	Fresh_week0	10	999	8.063	0.010	0.015
**Conditioned_week6**	Conditioned_week9	10	999	9.131	0.016	0.016
**Conditioned_week6**	Fresh_week0	10	999	12.405	0.015	0.016
**Conditioned_week9**	Fresh_week0	10	999	59.515	0.010	0.015

Table S5. Pairwise comparisons between substrate types at each timepoint based on the weighted UniFrac index. All p-values were calculated with the PERMANOVA test with 999 permutations.
